# Draft Genome Sequence of Lactococcus lactis subsp. lactis W8, a Potential Nisin-Producing Starter Culture for Indian Traditional Fermented Milk (Dahi)

**DOI:** 10.1128/MRA.01305-18

**Published:** 2018-12-13

**Authors:** Suranjita Mitra, Bidhan Chandra Mukhopadhyay, Tawsif Ahmed Kazi, Rajarshi Bhattacharya, Sukhendu Mandal, Swadesh Ranjan Biswas

**Affiliations:** a Department of Botany, Visva-Bharati, Santiniketan, West Bengal, India; b Department of Microbiology, University of Calcutta, Kolkata, India; University of California, Riverside

## Abstract

Dahi is a traditional Indian fermented milk consumed regularly as part of the diet because of its palatability and health benefits. Here, we report the draft genome sequence of a unique strain of Lactococcus lactis subsp. lactis, W8, a lactic acid bacterium that produces nisin while fermenting milk to dahi.

## Abstract (precis)

Dahi is a traditional Indian fermented milk consumed regularly as part of the diet because of its palatability and health benefits. Here, we report the draft genome sequence of a unique strain of Lactococcus lactis subsp.

## ANNOUNCEMENT

Lactococcus lactis is a food-grade Gram-positive lactic acid bacterium widely used in the dairy industry for the production of cheese. Certain L. lactis strains are reported to produce nisin, an antibacterial peptide commonly used as a natural food preservative (1). We reported a nisin-producing bacterium from naturally fermented milk (2), which was identified as L. lactis subsp. lactis W8 by biochemical and 16S rRNA gene sequence analysis (99% identity with L. lactis subsp. lactis). The uniqueness of this strain lies in its ability to produce nisin while fermenting milk to dahi (pH 4.2) within 6 h (3). Dahi displays antibacterial activity against food spoilage and pathogenic bacteria (3). The strain W8 grows and produces nisin even in 20-times-diluted skim milk (4). Thus, the strain W8 has improved characteristics for the economic production of nisin and fermented milk of safe quality (3). It survives at pH 3.0 and grows in the presence of bile (5). To explore the technological traits relevant to milk fermentation and the probiotic potential, the L. lactis W8 genome was sequenced.

Lactococcus lactis W8 was grown overnight at 37°C in tryptone, glucose, and yeast extract (TGE) broth, all at 1% (pH 6.5) (6), and genomic DNA was prepared using Qiagen Genomic-tip 100/G. The paired-end libraries were prepared using an Illumina TruSeq Nano DNA library prep kit and sequenced on a NextSeq 500 instrument with 2 × 150-bp chemistry at Eurofins Genomics India Pvt. Ltd. The data generated were processed to obtain high-quality reads using Trimmomatic v0.35 (7), quality checked using FastQC v0.11.7 (8), and *de novo* assembled using Velvet v1.2.10 (9) with default parameters. The contigs were scaffolded using SSPACE-standard v3.0 (10) with default parameters, generating 25 scaffolds (2,439,471 bp; GC content, 34.9%; *N*_50_, 147,833 bp) from 4,116,876 paired-end reads. Gene prediction and functional annotation were done using Blast2GO 5 (11) and the Rapid Annotations using Subsystems Technology (RAST) server (12). A total of 2,444 coding sequences with 58 RNA genes were annotated in the genome of W8. A cluster of 11 genes responsible for nisin production was annotated using BAGEL3 (13). The annotation predicted a number of milk utilization genes encoding *lacG*, *lacZ*, lactose-specific transporters, regulators, several proteinases, including Clp, peptidases, and oligopeptide and amino acid transporters. Unlike the reported dairy L. lactis strain (14), the biosynthetic potential of W8 for histidine, leucine, isoleucine, and valine was predicted using BlastKOALA (15), which might facilitate rapid growth in milk deficient in these free amino acids.

The potential of W8 to survive and persist in the gastrointestinal (GI) tract is predicted by the presence of two cholylglycine hydrolase genes, bile and acid stress resistance genes, and two adhering genes encoding fibronectin/fibrinogen-binding proteins. Further insight into the genome will create a new possibility for the production of novel probiotic dairy products with a built-in preservative.

### Data availability.

This whole-genome shotgun project has been deposited at DDBJ/ENA/GenBank under the accession number QYRO00000000. The version described in this paper is the first version, QYRO01000000. Raw sequencing data have been deposited in the NCBI Sequence Read Archive (16) database under the accession number SRR7868294.
